# Reduced-port robotic pancreaticoduodenectomy with optimized surgical field deployment: early results of single-site plus-two ports method

**DOI:** 10.1007/s00464-024-11097-y

**Published:** 2024-07-24

**Authors:** Riki Ninomiya, Masahiko Komagome, Satoru Abe, Shohei Maruta, Shinichi Matsudaira, Noriki Okada, Kazuhiro Mori, Rihito Nagata, Takehiro Chiyoda, Akifumi Kimura, Nobuyuki Takemura, Akira Maki, Yoshifumi Beck, Ching-Lung Hsieh, Cheng-Ming Peng

**Affiliations:** 1 Department of Hepato-Biliary-Pancreatic Surgery and Pediatric Surgery, Saitama Medical Center, Saitama Medical University, 1981, Kamoda, Kawagoe, Saitama 350-8550 Japan; 2 Hepato-Biliary-Pancreatic Division and Artificial Organ and Transplantation Division, Department of Surgery, Graduate School of Medicine, The University of Tokyo, Tokyo, Japan; 3 Da Vinci Minimally Invasive Surgery Center, Chung Shan Medical University Hospital, No.110, Sec 1, Chien-Kuo N. Rd., Taichung, 40201 Taiwan

**Keywords:** Minimally invasive pancreatectomy, Reduced-port robotic surgery, Robotic pancreaticoduodenectomy, Robotic surgical procedures, Single-port pancreatoduodenectomy

## Abstract

**Background:**

The adoption of Robotic Pancreaticoduodenectomy (RPD) is increasing globally. Meanwhile, reduced-port RPD (RPRPD) remains uncommon, requiring robot-specific techniques not possible with laparoscopy. We introduce a unique RPRPD technique optimizing surgical field exposure.

**Methods:**

Our RPRPD utilizes a single-site plus-two ports technique, facilitated by a single-port platform through a 5-cm incision. The configuration of robotic arms (arm1, arm2, arm3, and arm4) were strategically designed for optimal procedural efficiency, with the arms2 and arm3, alongside the assistant trocar, mounted on the single-port platform, while the arms1 and arm4 were positioned laterally across the abdomen. Drainage was established via channels created at the arm1 and arm4 insertion sites. A “gooseneck traction” was principally employed with the robotic instrument to prop up the specimen rather than grasp, improving the surgical field’s visibility and access. Clinical outcomes of patients who underwent RPRPD performed between August 2020 and September 2023 by a single surgeon across two centers in Taiwan and Japan were reviewed.

**Results:**

Fifty patients underwent RPRPD using the single-site plus-two ports technique. The gooseneck traction technique enabled goodsurgical field deployment and allowed for unrestricted movement of robotic arms with no collisions with the assistant instruments. The median operative time was 351 min (250–488 min), including 271 min (219–422 min) of console time and three minutes (2–10 min) of docking time. The median estimated blood loss was 80 mL (1–872 mL). All RPRPD procedures were successfully performed without the need for conversion to open surgery. Postoperative major morbidity (i.e., Clavien-Dindo grade ≥ IIIa) was observed in 6 (12%) patients and median postoperative hospital stay was 13 days.

**Conclusions:**

The single-site plus-two ports RPRPD with the gooseneck traction proves to be a safe, feasible option, facilitating surgical field visibility and robotic arm maneuverability.

**Graphical Abstract:**

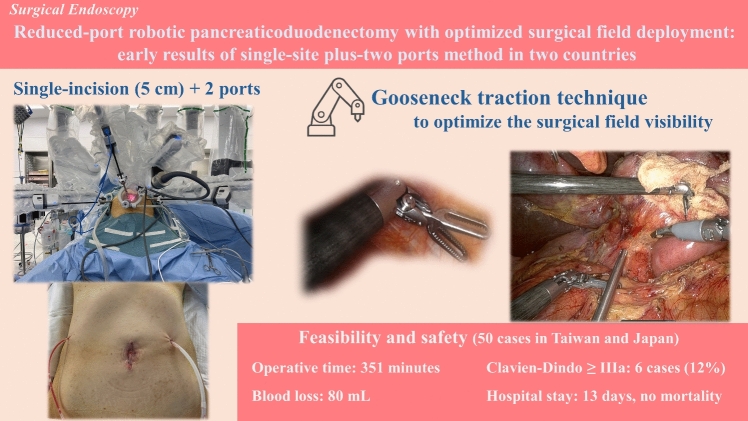

**Supplementary Information:**

The online version contains supplementary material available at 10.1007/s00464-024-11097-y.

The application of minimally invasive pancreatectomy (MIP) has gradually spread worldwide in the past few decades. In laparoscopic surgery, laparoscopic distal pancreatectomy has been accepted globally, as its feasibility and safety have been demonstrated compared with open surgery [1, 2], whereas laparoscopic pancreaticoduodenectomy (PD) is still challenging because of the complex procedures of both dissection and reconstruction [3–5]. The widespread use of robotic surgery has enabled innovative procedures such as motion scaling, reduced tremor, and internal articulation of the wrist. In addition to these features, robotic pancreaticoduodenectomy (RPD) is becoming accepted because it includes three-dimensional visualization and up to seven degrees of freedom [6]. On the other hand, reduced-port surgery (RPS) is gaining recognition in parallel with the aforementioned advances and is expected to reduce invasion compared to conventional laparoscopic surgery [7, 8]. However, RPS has technical limitations, including the formation of triangulation and instrument collisions. Robotic surgery has the potential to overcome these limitations, and the advantages of robotics over RPS have been reported in various fields [9–11]. For MIP, reduced-port robotic distal pancreatectomy (RPRDP) has been reported in a several institutions [12, 13]; however, few surgeons have been performing reduced-port robotic pancreaticoduodenectomy (RPRPD). To achieve safe execution of RPRPD, the surgical field deployment requires a different perspective from laparoscopic surgery due to the unique characteristics of the robot. This study retrospectively demonstrates the clinical outcomes of RPRPD with an optimized surgical field deployment technique by a single surgeon across two centers in Taiwan and Japan, focusing on the feasibility and safety of RPRPD.

## Materials and methods

### Patient selection and study design

The present study is a prospective single-surgeon experience, encompassing consecutive RPRPD cases performed by the same surgeon (RN) across institutions in Japan and Taiwan, spanning from August 2020 to September 2023. This study adheres to the observational cohort studies guidelines as outlined in STROBE and its extensions. The interventions conducted in Taiwan coincided with the author’s clinical fellowship at the Minimally Invasive Surgery Center, Chung Shan Medical University Hospital (from August 2020 to July 2021). Prior to performing RPRPD, the surgeon had conducted four RPDs using the multi-port technique. RPRPD was implemented for all patients with pancreatic head and periampullary lesions, whether benign or malignant, and those who did not require reconstruction of the portal vein (PV) or major arteries. Additionally, patients with a history of major upper abdominal surgeries, such as operations for malignant tumor, were not considered suitable candidates for RPRPD. All RPRPDs have been performed using Da Vinci Xi or X system® (Intuitive Surgical, Sunnyvale, CA, USA). Patient demographics and perioperative outcomes, including operation time, estimated blood loss, and postoperative complications were retrospectively reviewed. Postoperative complications were assessed according to the Clavien–Dindo classification (C–D) [14], and postoperative pancreatic fistula (POPF) was graded according to the guidelines of the International Study Group on Pancreatic Surgery [15]. This study was approved by the Review Board of Saitama Medical Center, Saitama Medical University (approval number: Sou2021-121) in Japan and the Research Ethic Committee of Chung Shan Medical University Hospital (approval number: CS1-20103) in Taiwan.

### Statistical analysis

Patient backgrounds and perioperative outcomes were analyzed using JMP Pro 16 (SAS Institute Inc., Cary, NC, USA). Categorical variables, expressed as counts and percentages, were compared using either the chi-square test or Fisher’s exact test, depending on the number of cases. Continuous variables, presented as median values with their minimum and maximum, were compared using the Mann–Whitney *U* test. Furthermore, the learning curves for perioperative outcome were calculated using the cumulative sum (CUSUM) control chart.

### Surgical technique

**Fig. 1:**
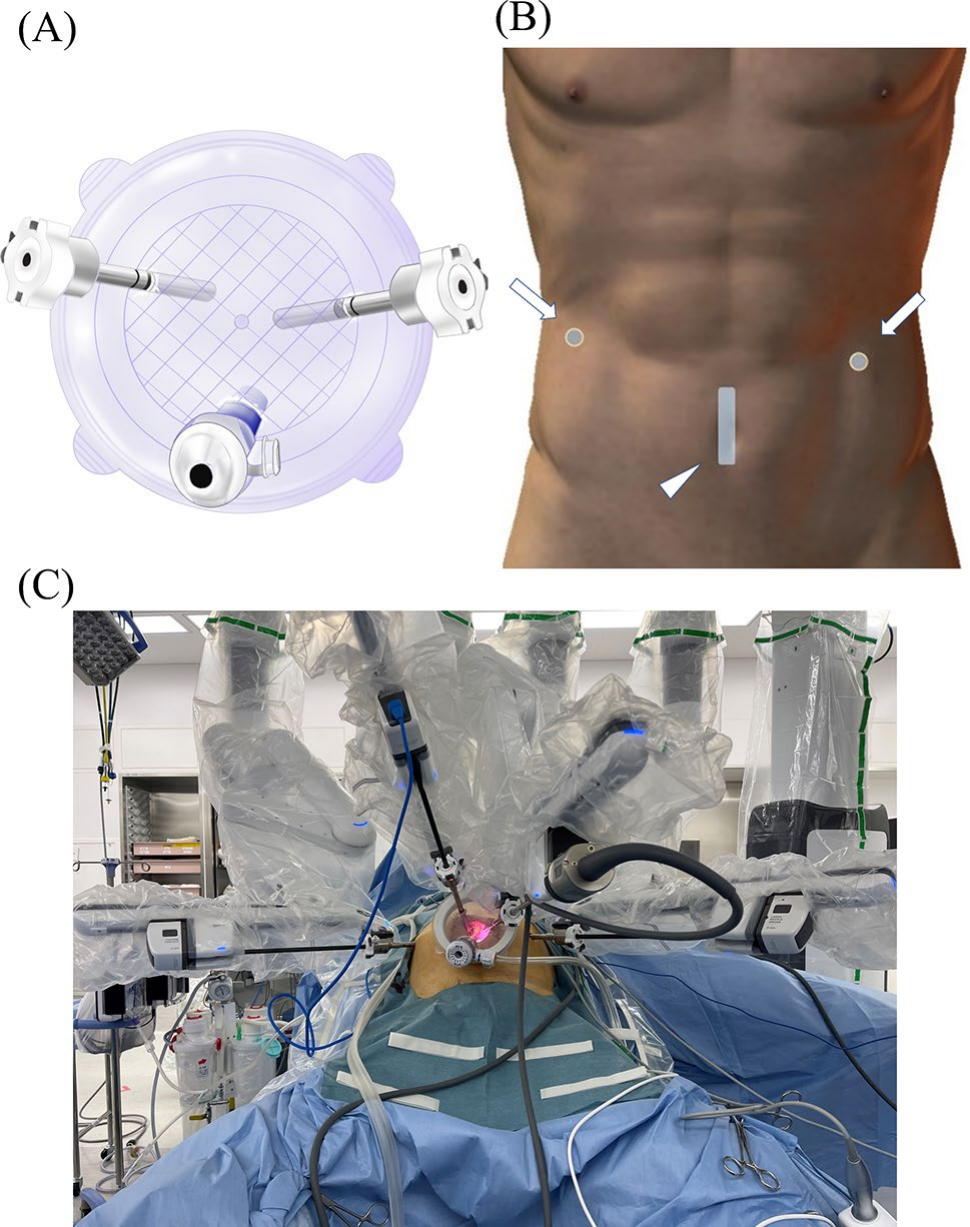
Robot and trocars setup. **A** The two robotic trocars and an assistant trocar installed on the single-port platform. **B** A schema of the incision. The single-port platform was installed on the 5 cm measured transumbilical incision (triangular arrow), with the robotic trocars placed on both sides of the abdomen (arrow). **C** Robotic arms are placed as a single-site plus-two ports technique

The patient was positioned in a 15-degree reverse Trendelenburg posture with legs separated. Prior to the procedure, a 5 cm transumbilical incision was created, onto which the LapBase 110 MA® (LAGIS, Taichung, Taiwan) was affixed, serving as the single-port platform. The patient cart was set from the left cranial side, and the robotic arm NO.2 (arm2), NO.3 (arm3), and a 12 mm assistant trocar were installed on the single-port platform (Fig. 1A). The three-dimension camera was set on the arm3. As shown in Fig. 1B, the robotic arms NO.1 (arm1) and NO.4 (arm4) were then placed on the right and left side of the abdomen. Consequently, a console procedure was started as a single-site plus-two ports (SP2) technique (Fig. 1C, VideoS1). The docking time was defined as the time from the patient cart roll-in to the start of the console procedure.

The arm1 and arm2 were installed with Cadiere forceps® and Force bipolar® (Intuitive Surgical, Sunnyvale, CA, USA), respectively. The arm4 was basically fitted with a cutting device, where a Permanent cautery hook®, SynchroSeal®, or Harmonic ACE® was used depending on the situation. An artery and a thick branch of the PV or the superior mesenteric vein (SMV) were clamped using Hem–o–lok® clips (Teleflex Medical, USA) with a Large clip applier®.

**Fig. 2:**
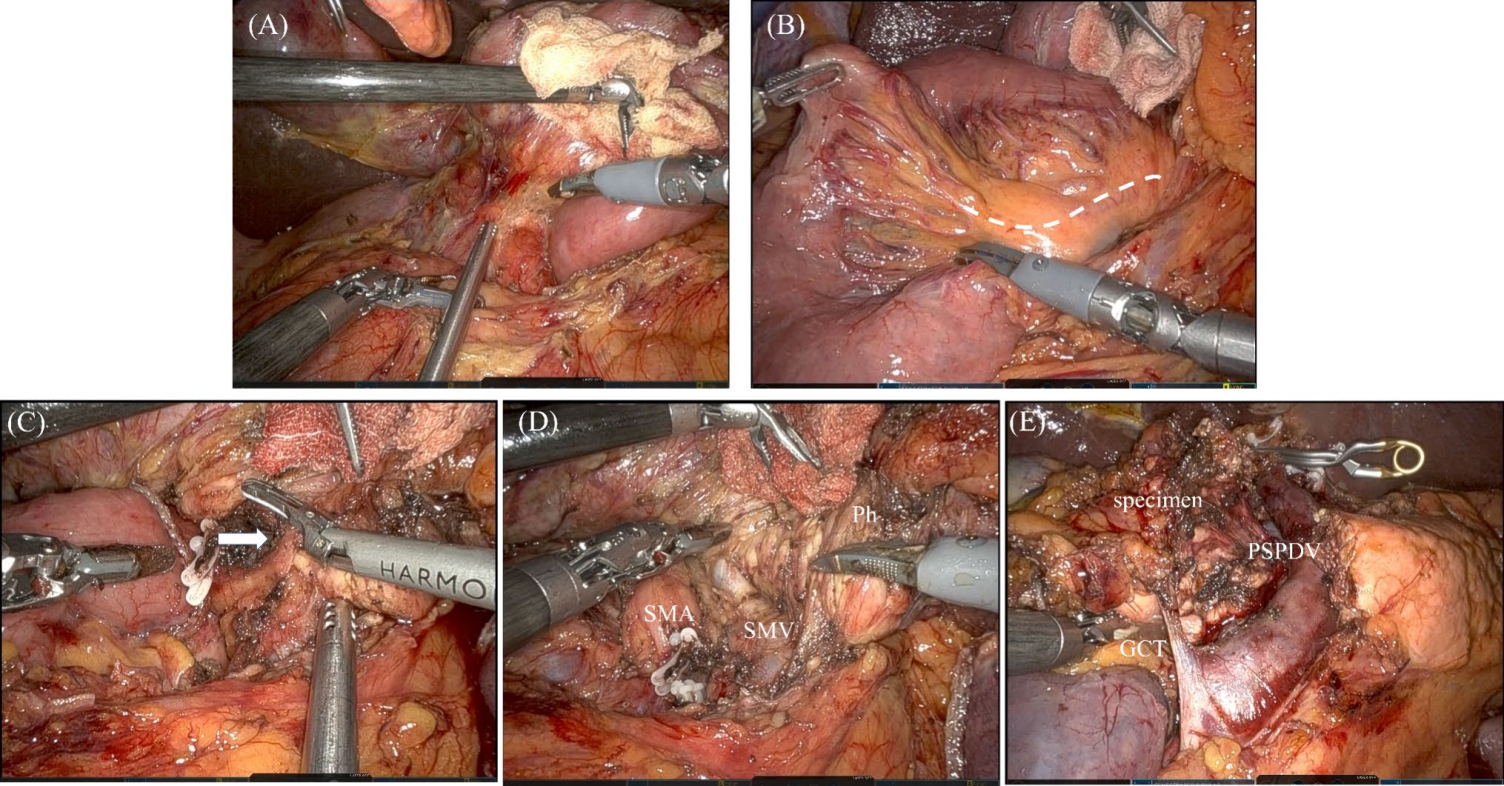
Intraoperative pictures. Intraoperative pictures in resection of reduced-port robotic pancreaticoduodenectomy. **A** A gooseneck traction in the Kocher’s maneuver. **B** The jejunum was pulled out to the right side then the mesentery of the jejunum was divided along the first jejunal artery (dotted line). **C** The IPDA (arrow) was identified and clamped. **D** The plexus around the pancreatic head was dissected and the posterior wall of the SMV was exposed. **E** Following transecting the pancreas on the SMV, the specimen was taken out with only some branches resected. *GCT* gastrocolic trunk, *IPDA* inferior pancreaticoduodenal artery, *Ph* pancreatic head, *PSPDV* posterior superior pancreatoduodenal vein, *SMA* superior mesenteric artery, *SMV* superior mesenteric vein

In the robotic procedure, our approach primarily involves manipulating the surgical fields through traction rather than direct grasping with a robotic arm. To facilitate broader countertraction, the specimen is elevated using the instrument’s head, positioned in a flexed manner reminiscent of a gooseneck shape. This technique is referred to as "gooseneck traction" (Fig. 2A, Video S2). In approaching the Superior Mesenteric Artery (SMA), we have adopted the right lateral approach as previously reported by our team [16].

As an initial step in robotic procedure, the gallbladder was grasped and lifted using an internal organ retractor (AESCULAP®, B. Braun SE, Melsungen, Germany) to elevate the liver. The Kocher’s maneuver was performed with the gooseneck traction to mobilize the pancreatic head and the duodenum from the retroperitoneum. The stomach was transected using an automated stapler, and the jejunum was then pulled to the right side through the opened part of the ligament of Treitz (Fig. 2B). The mesentery of the jejunum was divided along the first jejunal artery (J1A), and the jejunum was then transected. Subsequently, the specimen was rotated further clockwise and was held with the gooseneck traction to superficialize the SMA to the rightmost side. Then, the inferior pancreaticoduodenal artery and J1A were clamped (Fig. 2C). Subsequently, the plexus of the pancreatic head was divided and the posterior side of the SMV was exposed (Fig. 2D). The stump of the stomach was retracted to the right side using the internal organ retractor to gain access to the hepatoduodenal ligament. The liver was then elevated with the gooseneck traction. The hepato-duodenal ligament dissection was performed, and the gastroduodenal artery was divided. The gallbladder was then removed, and the bile duct was transected. After transecting the pancreas on the SMV using the Harmonic scalpel®, the specimen was removed through the single-site incision with resection of some branches from the SMV and PV (Fig. 2E, Video S3).

**Fig. 3:**
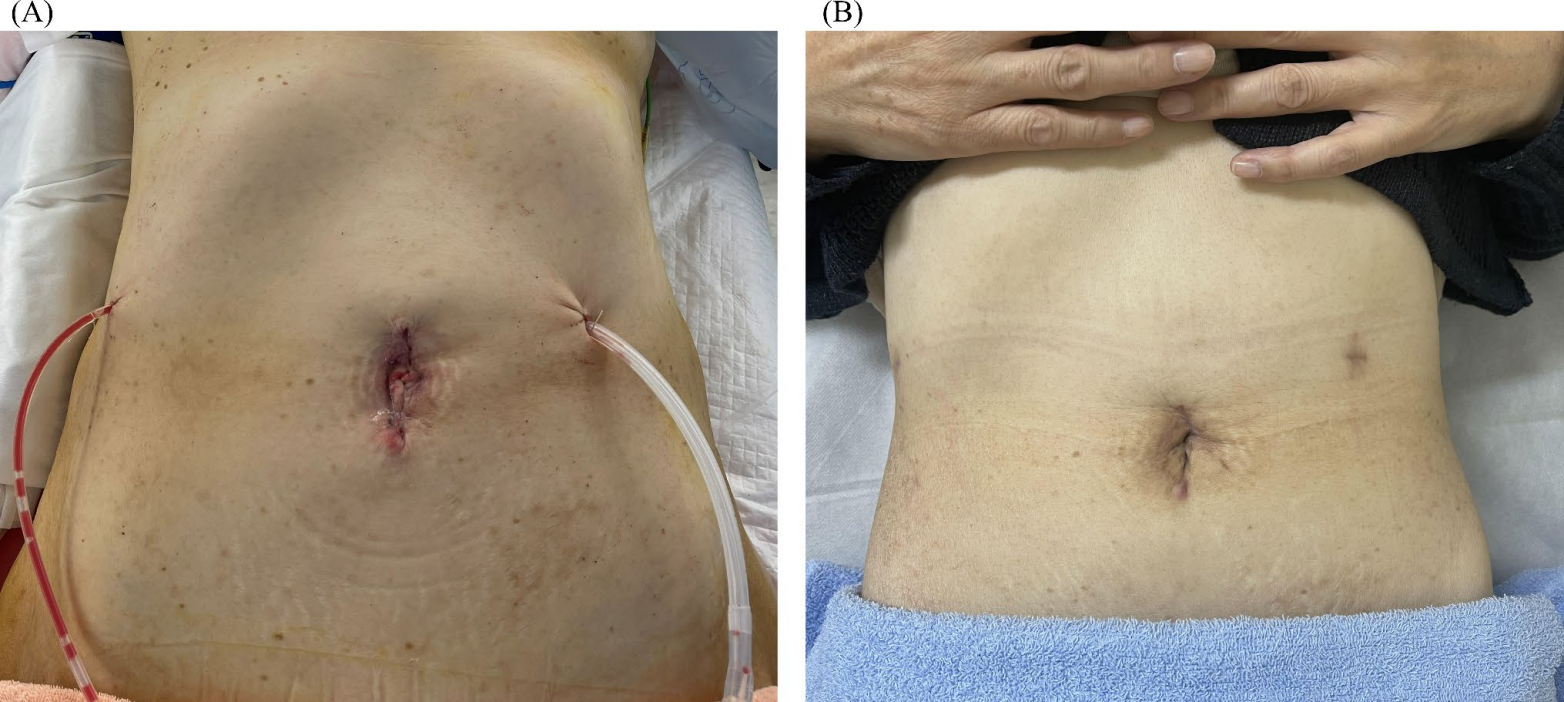
Postoperative wounds. The wound after reduced-port robotic pancreaticoduodenectomy. **A** 5 cm diameter incision and two drainage tubes from both sides. **B** One month after surgery

While performing the reconstruction procedure, the liver was lifted using the gooseneck traction. Firstly, a pancreatojejunostomy was performed using the modified Blumgart anastomosis [17], and six interrupted sutures were used for the anastomosis of the main pancreatic duct with the jejunal mucosa. Choledochojejunostomy (CJ) was primarily performed using a single needle for a continuous suture, with interrupted sutures being employed as appropriate in cases where the bile duct was of a smaller diameter. Gastrojejunostomy was completed either robotically or via the incision of single-port platform using an automated stapler. Drain tubes were inserted through the wounds at the arm1 and arm4, and the procedure was concluded (Fig. 3A). Figure 3B shows the wound one month after RPRPD.

## Results

We reviewed 50 consecutive patients who underwent RPRPD using the SP2 technique for various indications in Taiwan and Japan, encompassing 36 malignant and 14 benign tumors (Table 1). Among the malignant cases, the distribution was as follows: pancreatic invasive ductal adenocarcinoma (*n* = 17), adenocarcinoma of the ampulla of Vater (*n* = 8), bile duct cancer (*n* = 7), intraductal papillary mucinous carcinoma (*n* = 3), and duodenal cancer (*n* = 1). The median age of the patients was 57 years. The sex distribution was balanced with 26 females (52%) and 24 males (48%). The median body mass index (BMI) was 24.8 kg/m^2^. As shown in Table 2, the median operative time was 351 min (range, 250–488 min), including 271 min (range, 219–422 min) of the console time and three minutes (range, 2–10 min) of the docking time. The median estimated blood loss was 80 mL (range, 1–872 mL). All RPRPD procedures were successfully performed without the need for conversion to open surgery; however, one patient (2%) necessitated the placement of an additional trocar due to hemorrhage at the PV. In all cases of malignant tumors, complete resection without any residual pathology was achieved. Grade B of POPF was observed in 5 patients (10%). Complications classified as Clavien-Dindo Grade IIIa or higher were recorded in 6 patients (12%), with POPF occurring in three patients (6%), and bile leakage, pneumothorax, and postoperative bleeding each observed in one patient (2%). Notably, there were no reported cases of Grade C of POPF. It is worth mentioning that the patients who experienced postoperative bleeding did so due to arterial dissection caused by the use of an energy device. The median duration of postoperative hospitalization was 13 days, with a range from 7 to 67 days, and there were no instances of mortality observed.

**Table 1:** Patient backgrounds

	*n* = 50
Age, year	57 (28–84)
Sex female/male, *n*	26/24
BMI, kg/m^2^	24.8 (17.6–38.8)
Malignant/Benign, *n*	36/14
PDAC	17
Ampulla of Vater cancer	8
Bile duct cancer	7
IPMC	3
Duodenal cancer	1
IPMN	7
pNEN	3
GIST	3
MCN	1

*BMI* body mass index, *GIST* gastrointestinal stromal tumor, *IPMC* intraductal papillary mucinous carcinoma, *IPMN* intraductal papillary mucinous neoplasm, *MCN* mucinous cystic neoplasm, *PDAC* pancreatic invasive ductal adenocarcinoma, *pNEN* pancreatic neuroendocrine neoplasm, *POPF* postoperative pancreatic fistula

**Table 2:** Perioperative outcomes

	*n* = 50
Operation time, minutes (range)	351 (250–488)
Console time, minutes (range)	271 (219–422)
Docking time, minutes (range)	3 (2–10)
Estimated blood loss, mL (range)	80 (1–872)
Additional trocar requirement, *n* (%)	1 (2%)
Conversion to open surgery, *n*	0
POPF grade B, *n* (%)	5 (10%)
Clavien-Dindo ≥ IIIa, *n* (%)	6 (12%)
POPF	3 (6%)
Bile leakage	1 (2%)
Pneumothorax	1 (2%)
Postoperative bleeding	1 (2%)
Postoperative hospital stay, day (range)	13 (7–67)
Mortality,* n*	0

*POPF* postoperative pancreatic fistula

**Fig. 4:**
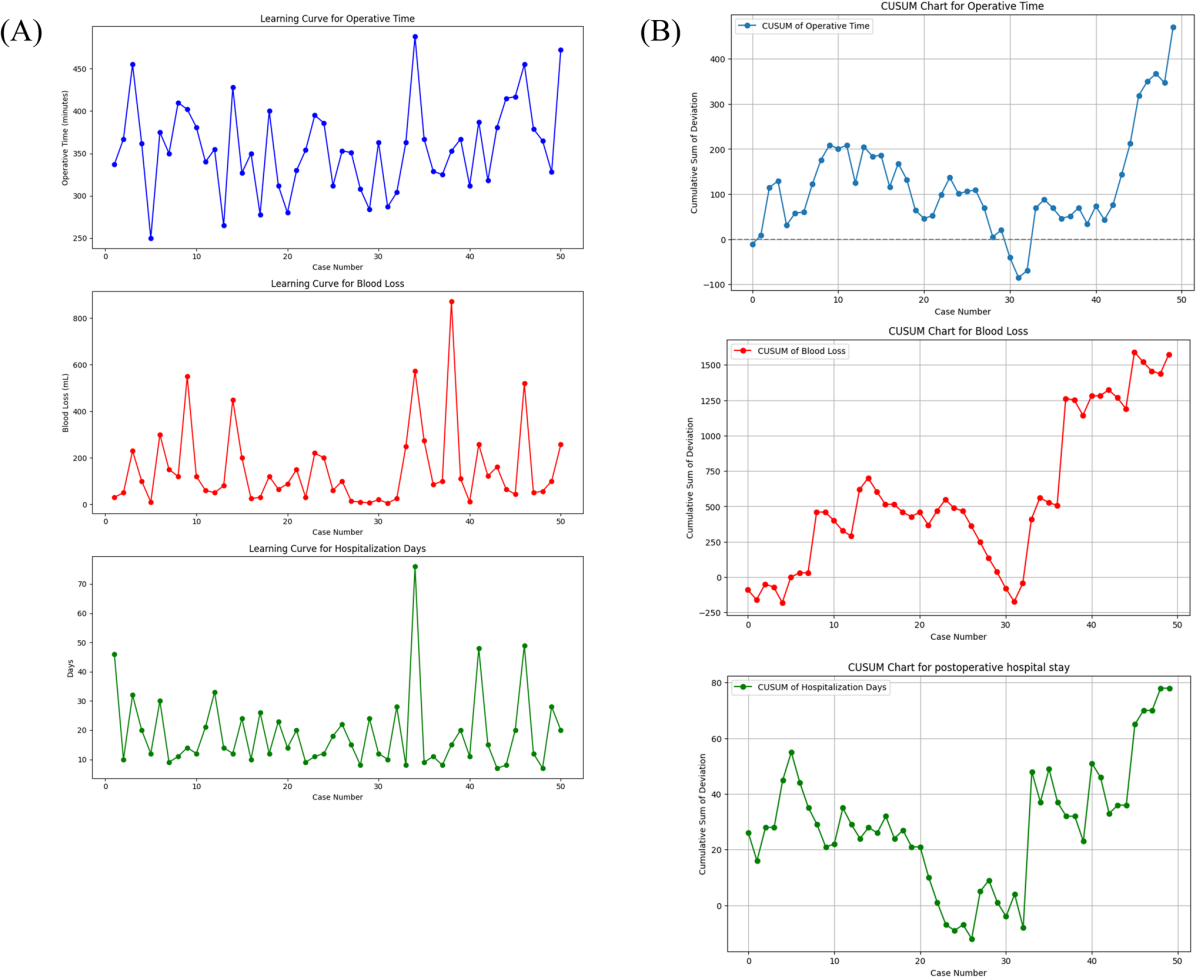
Learning curve for perioperative outcome. **A** Trends in the learning curve for operative time, blood loss, and postoperative hospital stay. **B** Cumulative sum charts for operative time, blood loss, and postoperative hospital stay. Abbreviations, CUSUM; cumulative sum

Temporal trends in the learning curve for operative time, blood loss, and postoperative hospitalization are illustrated in Fig. 4A. While initial observations may not suggest the attainment of a learning curve, CUSUM analysis conducted on operative time, blood loss, and postoperative hospitalization days, all showed a decrease in cumulative sums after the 20th case, followed by a gradual increase (Fig. 4B). No clear learning curve was observed for CR-POPF or postoperative complications.

## Discussion

Although RPD remains a technically complex procedure, our RPRPD employing the SP2 technique showed promising results in terms of operative time, blood loss, and postoperative complications. While these outcomes appear favorable when considered alongside those reported in previous systematic reviews and meta-analyses of RPD [4, 18–20], it is important to note that direct comparisons are limited by the lack of statistical analysis comparing our results directly with these studies. Therefore, we claim that our approach demonstrates feasibility and safety, and we look forward to further studies that could substantiate our findings with more robust statistical evidence. Importantly, our robot-specific technique, which involves the use of the gooseneck traction to optimize the surgical field of view, is not exclusive to RPRPD but is also applicable to conventional RPD. This is a significant finding that highlights the versatility and potential impact of our technique in improving surgical outcomes.

The implementation of the gooseneck traction technique in our robotic surgical procedures has effectively optimized the surgical field. This method enhances the inherent benefits of robotic surgery, such as improved stability, by allowing the console surgeon exclusive control over field deployment, thereby streamlining operations, and minimizing the risk of interference and potential harm to assistants from robotic arm movements. While robotic arm manipulation can sometimes hinder performance, this challenge is not unique to RPS but extends to conventional robotic techniques as well, underlining its general applicability. Additionally, in RPRPD, the proximity of robotic arms on the single-port platform restricts the ability of assistant surgeons to freely perform traction within the surgical field, further complicating the process. In gooseneck traction, the forceps are flexed in a manner resembling a gooseneck to elevate organs, rather than simply grasping them. This technique distributes tension across a larger surface area of the organ, rather than focusing it at a single point, thereby expanding the surgical field. Additionally, gauze may be inserted between the forceps and the organ to further extend the exposed area. Consequently, this method enables effective surgical field deployment using a single forceps, simplifying the procedure and enhancing efficiency. This method facilitates efficient execution and minimizes tissue injury risk, enhancing procedural safety and precision. Furthermore, the gooseneck traction has a significant effect on the surgical field deployment for approaching to the SMA, which is one of the most crucial parts of PD. Nagakawa et al. summarized four strategies for approaching the SMA in minimally invasive PD [21]. Our technique integrates and modifies the posterior and right approaches, facilitating a unique pathway to the SMA [16]. The gooseneck traction necessitates a more pronounced clockwise rotation compared to the traditional posterior approach, thereby exposing the targets more effectively. Through this approach, the gooseneck traction enables clear and efficient access to the SMA, positioned anteriorly, from a posterior viewpoint. This facilitates safe and precise arterial management. The surgeon had performed approximately 320 open PDs, including this approach, which likely facilitated a rapid adoption of the technique in RPD.

Regarding the learning curve, we have presented the results of CUSUM analysis for operative time, blood loss, and postoperative hospital stay, drawing on previously published studies [22]. These results suggest that stabilization and improvement of surgical techniques were observed after approximately 20 cases. However, deterioration in performance was noted after around the 40th case for each parameter. This decline may be attributed to factors such as proactive surgical intervention in cases with inflammatory complications such as cholangitis or pancreatitis, and the expanded application of surgery to advanced cancer cases, potentially introducing bias.

The first case of reduced-port robotic surgery in humans was reported in the urological field in 2009 [23], followed by several cases of gynecological and colorectal surgery during the same period [24, 25]. Although the application of robotic RPS has been spread to many fields, reduced-port robotic pancreatectomy was first reported by Kim et al. with RPRDP in 2017, a decade later than that in other fields [12]. Subsequently, Chiang et al. conducted the study on RPRPD, demonstrating pure single-site and single-site plus-one port RPD [26]. To our knowledge, this is the inaugural report of RPRPD utilizing the SP2 technique globally [27]. The inception of this innovative procedure was primarily motivated by the necessity for effective drainage. We contend that the drainage of regions superior to the pancreas and around the CJ is imperative, advocating for the placement of drains bilaterally. The SP2 technique enhances procedural safety by synchronizing arm2 with the camera, substantially reducing the risk of arm collisions. This synchronization allows for collision prevention comparable to that observed in multi-port RPD. Additionally, this technique, along with the unique arrangement of the single-port platform, naturally augments the distance between the robotic arms as they converge, ensuring a collision-free environment that facilitates precise manipulations by the surgeon at the console. The single-port platform employed permits the insertion of a trocar through a 5 cm incision, maintaining an optimal separation of at least 7 cm between the arm2, arm3, and assistant’s trocar, thereby ensuring unimpeded movement of the robotic arms and camera. This strategic placement is crucial for maintaining clear visualization throughout the procedure and minimizing potential interference with the assistant’s instruments. Furthermore, the initial establishment of a small laparotomy incision offers significant advantages, such as facilitating adhesiolysis of the intestines and other adhesions before the robotic procedure, and enabling the direct visual performance of gastrojejunostomy during reconstruction. This strategy has been instrumental in shortening the operative time and enhancing overall safety. The practicality and efficiency of the SP2 technique were underscored by a median docking time of just three minutes, highlighting its straightforward implementation.

This study faces several limitations. Initially, our experience with conventional multi-port RPD was limited, making it difficult to directly compare perioperative outcomes or fully articulate the benefits of RPRPD. This limitation restricts our capacity to conclusively evaluate the effectiveness of the gooseneck traction technique across both reduced-port and traditional RPD methods, rendering any comparative conclusions about its efficacy speculative at best. Additionally, the relatively low BMI of the patient cohort may introduce bias in the selection process for robotic procedures, suggesting that outcomes such as operative time and estimated blood loss might not be solely attributable to the method’s efficiency but also influenced by patient selection. Furthermore, a comparison between RPRPD and open PD should have been conducted. However, due to selection criteria, most open PD cases involved advanced pancreatic cancer requiring vascular resections such as of the PV or hepatic artery. Consequently, it was not feasible to adjust for background factors and perform a meaningful comparison. Given these considerations, the findings presented here should be viewed as preliminary. Future studies, particularly those involving multiple operators and centers, are essential to comprehensively assess the effectiveness of RPRPD and the gooseneck traction technique.

In conclusion, the SP2 technique using the gooseneck traction is a safe and feasible approach for RPRPD. The gooseneck traction enabled the optimization of wide surgical field deployment and could be a key process for successful RPRPD. Further studies are warranted to confirm the safety and efficacy of this approach in larger patient cohorts.

## Supplementary Information

Below is the link to the electronic supplementary material.

Supplementary file1 (MP4 30336 KB)–Robot and trocars setup. The setup for reduced-port robotic pancreaticoduodenectomy using the Single-Site Plus-2 technique

Supplementary file2 (MP4 199069 KB)–Gooseneck traction in Kocher’s maneuver. Kocher’s maneuver using a gooseneck traction technique in reduced-port robotic pancreaticoduodenectomy

Supplementary file3 (MP4 471763 KB)–Resection procedure of reduced-port robotic pancreaticoduodenectomy. Resection stage of reduced-port robotic pancreaticoduodenectomy. Abbreviation, GDA; gastroduodenal artery IPDA; inferior pancreaticoduodenal artery, SMA; superior mesenteric artery, SMV; superior mesenteric vein
